# Supplementary material for: Static palpation ain’t easy: Evaluating palpation precision using a topographical map of the lumbar spine as a reference
Source: PLoS One. 2024 May 30;19(5):e0304571. doi: 10.1371/journal.pone.0304571 (PMC11139336; doi:10.1371/journal.pone.0304571)
Supplement: S1 File — (DOCX) [file pone.0304571.s001.docx]

**Supporting information 1**

A video showing the procedure of measuring the stiffness can be found at: <http://smerteforskning.dk/qst/video/vTrack.mp4> [Danish narration].
