# Supplementary material for: Static palpation ain’t easy: Evaluating palpation precision using a topographical map of the lumbar spine as a reference
Source: PLoS One. 2024 May 30;19(5):e0304571. doi: 10.1371/journal.pone.0304571 (PMC11139336; doi:10.1371/journal.pone.0304571)

**Supporting information 2 - The precision of palpation**

Linear mixed models with differences in spinous process location as the dependent variable and time interacting with spinous process level as the independent variable and participant as the random error

**Vertebral level**

**S2 – Table 1: Time assessment interacting with spinous process level**


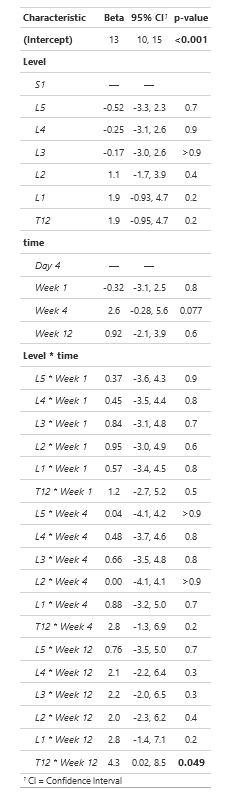


**S2 – Table 2: Spinous process level**


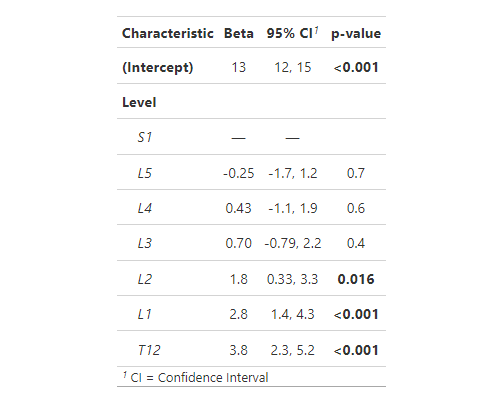


**S2 – Table 3: Time assessment**


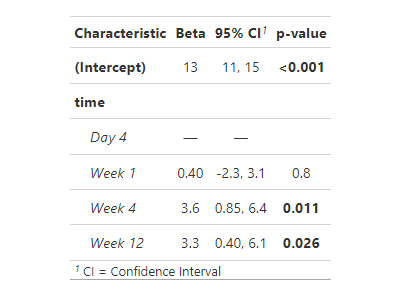


**Length of the lumbar spine**

**S2 – Table 4: Time assessment**


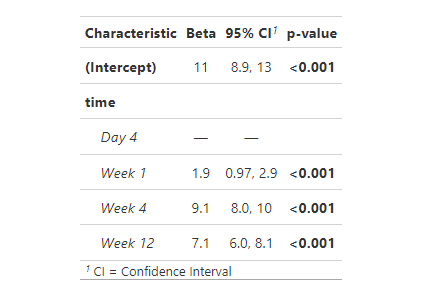

Supplement: S2 File — (DOCX) [file pone.0304571.s002.docx]
