# Supplementary material for: Static palpation ain’t easy: Evaluating palpation precision using a topographical map of the lumbar spine as a reference
Source: PLoS One. 2024 May 30;19(5):e0304571. doi: 10.1371/journal.pone.0304571 (PMC11139336; doi:10.1371/journal.pone.0304571)

**Supporting information 3**

Univariable linear regression with the summarized mean difference (across time and spinous process level) as the dependent factor and the descriptive data as the independent factor.

**Patient factor**

**S3 – Table 1: Vertebral level**


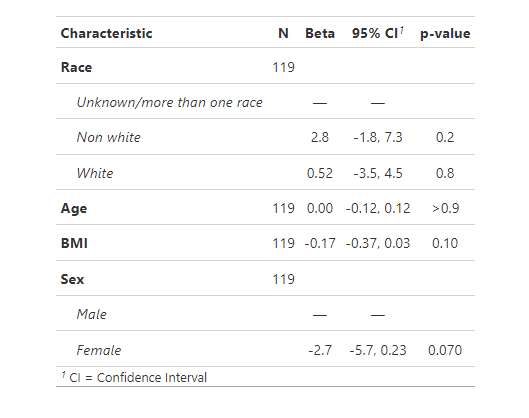


**S3 – Table 2: Length of the lumbar spine**


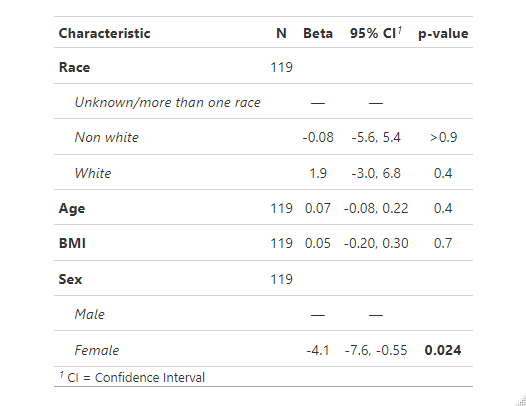


**Clinician info**

**S3 – Table 3: Vertebral level**


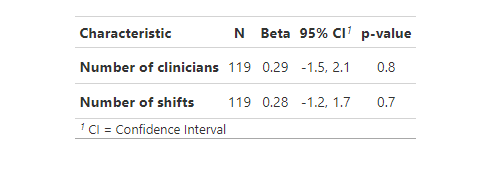


**S3 – Table 4: Length of the lumbar spine**


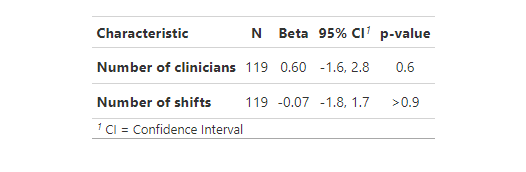

Supplement: S3 File — (DOCX) [file pone.0304571.s003.docx]
