# Supplementary figures and images for: Static palpation ain’t easy: Evaluating palpation precision using a topographical map of the lumbar spine as a reference
Source: PLoS One. 2024 May 30;19(5):e0304571. doi: 10.1371/journal.pone.0304571 (PMC11139336; doi:10.1371/journal.pone.0304571)

**Supporting information 4**

**S4 – Figure 1**


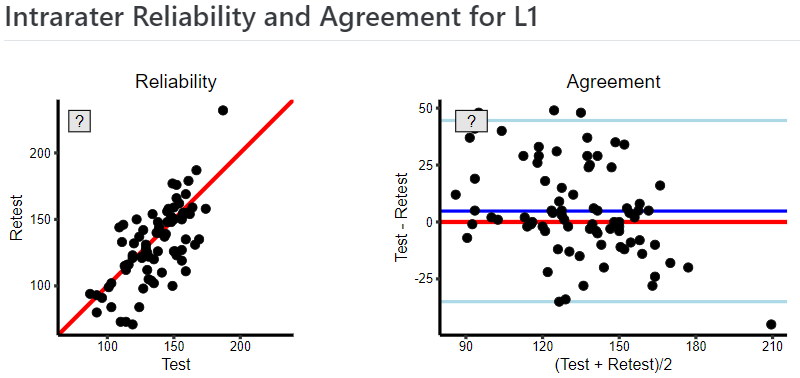


**S4 – Figure 2**


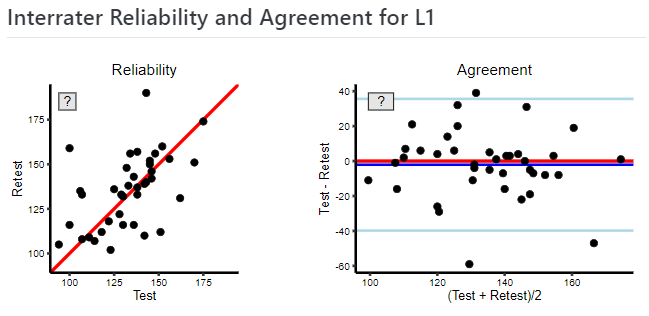


**S4 – Figure 3**


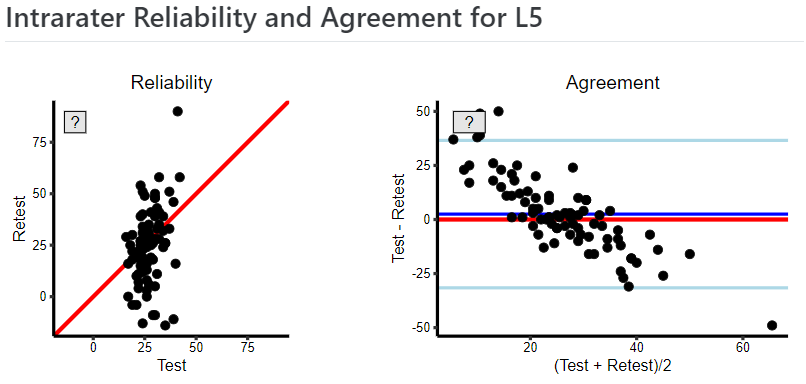


**S4 – Figure 4**


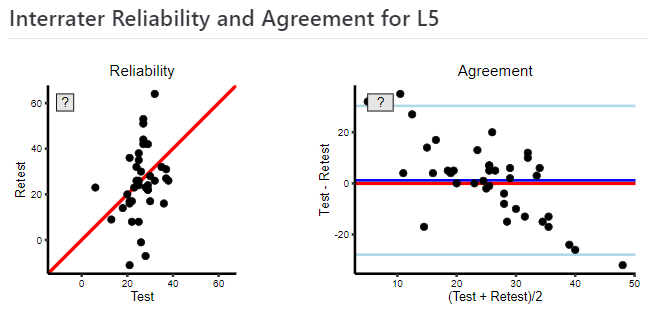


**S4 – Figure 5**


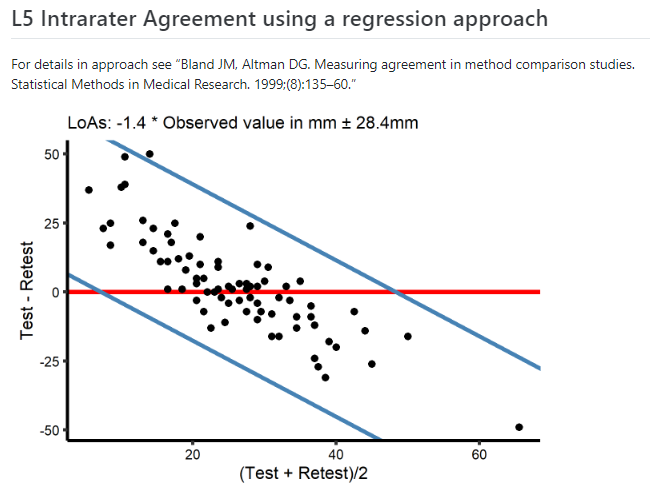


**S4 – Figure 6**


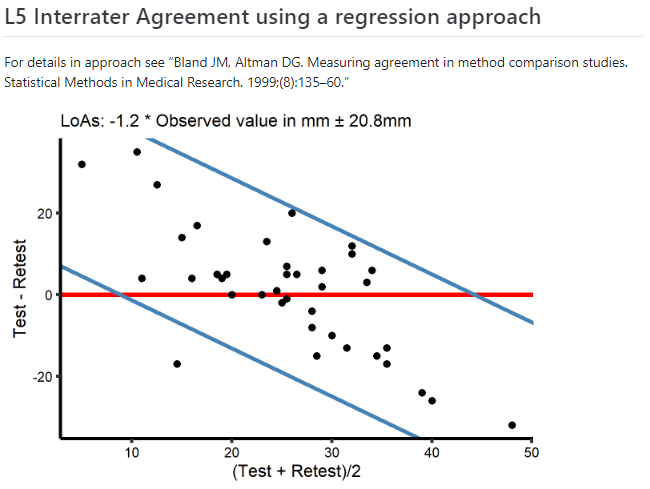

Supplement: S4 File — (DOCX) [file pone.0304571.s004.docx]
