# Supplementary material for: Static palpation ain’t easy: Evaluating palpation precision using a topographical map of the lumbar spine as a reference
Source: PLoS One. 2024 May 30;19(5):e0304571. doi: 10.1371/journal.pone.0304571 (PMC11139336; doi:10.1371/journal.pone.0304571)

**Supporting information 5**

Univariable linear regression with precision estimates as the dependent variable and the total number of clinicians who provided palpation data and the number of shifts in clinicians (i.e., going from Clinician A to B is one shift, and going from B to A would be a second shift).

**Associations with clinician factors**

**S5 – Table 1: Vertebral level**


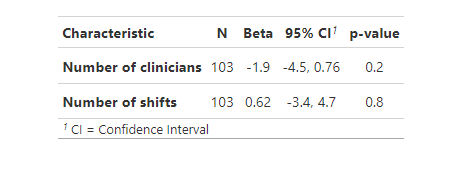


**S5 – Table 2: Length of the lumbar spine**


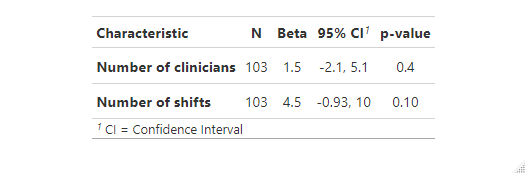

Supplement: S5 File — (DOCX) [file pone.0304571.s005.docx]
