# Supplementary material for: Static palpation ain’t easy: Evaluating palpation precision using a topographical map of the lumbar spine as a reference
Source: PLoS One. 2024 May 30;19(5):e0304571. doi: 10.1371/journal.pone.0304571 (PMC11139336; doi:10.1371/journal.pone.0304571)

**Supplementary material 6**

Linear mixed models with differences in spinous process location as the dependent variable and time interacting with spinous process level as the independent variable and participant as the random error. Sensitivity analysis using week 1 instead of week 0 as the reference

**Vertebral level**

**S6 – Table 1: Time assessment interacting with spinous process level**


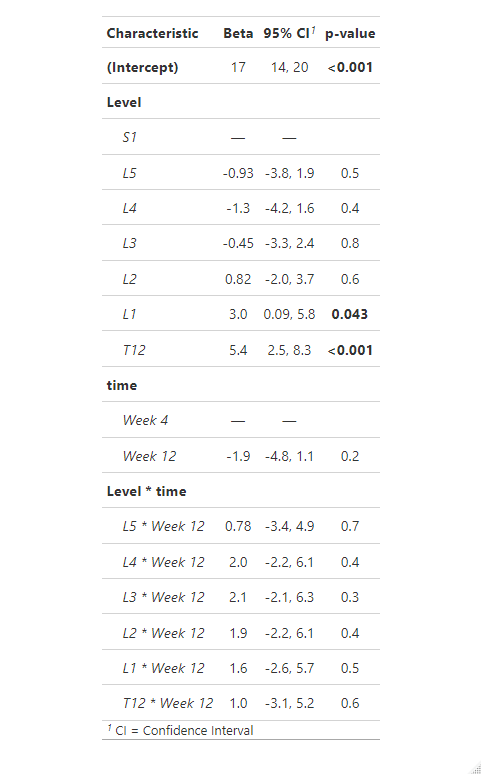


**S6 – Table 2: Spinous process level**


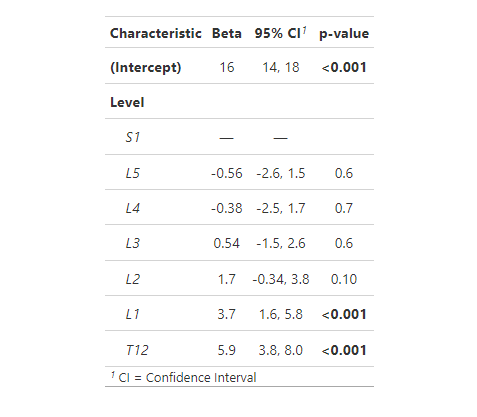


**S6 – Table 3: Time assessment**


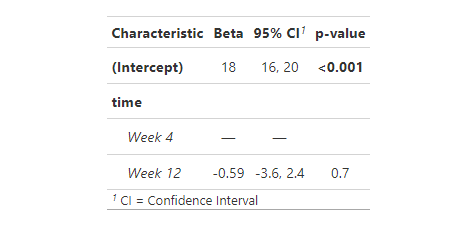


**Length of the lumbar spine**

**S6 – Table 4: Time assessment**


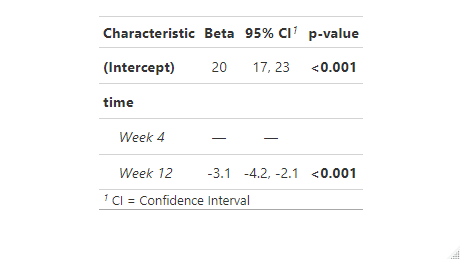


Univariable linear regression with the summarized mean difference (across time and spinous process level) as the dependent factor and the descriptive data as the independent factor. Sensitivity analysis using week 1 instead of week 0 as the reference

**Patient factor**

**S6 – Table 5: Vertebral level**


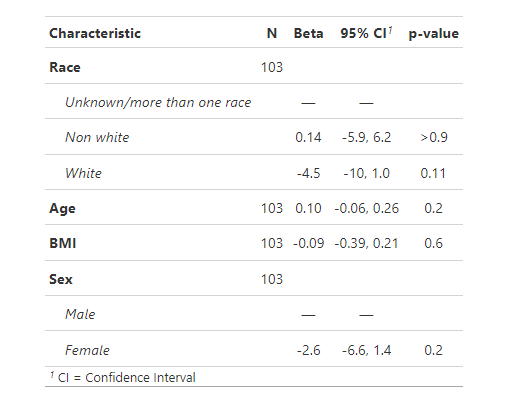


**S6 – Table 6: Length of the lumbar spine**


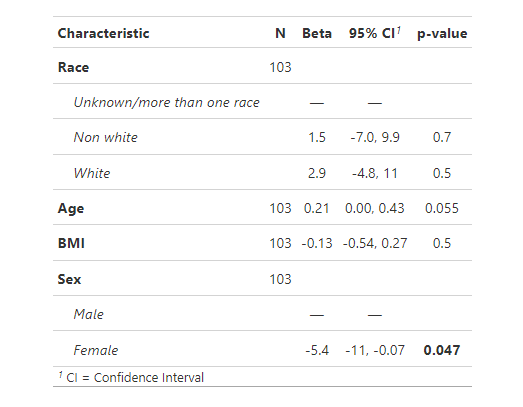

Supplement: S6 File — (DOCX) [file pone.0304571.s006.docx]
